# S-Allyl Cysteine Accumulation Depends on Temperature and Aging Duration of an *Allium* Organosulfur Compound Blend (*Allium sativum*)

**DOI:** 10.5812/ijpr-167972

**Published:** 2026-08-18

**Authors:** Shiva Shamshiri, Mehran Mirabzadeh Ardekani, Hamid Reza Moghimi, Ehsan Sadeghnezhad

**Affiliations:** 1 Department of Traditional Pharmacy, Faculty of Pharmacy, Tehran University of Medical Science, 14176-14411, Tehran, Iran; 2 Department of Pharmaceutics and Pharmaceutical Nanotechnology, School of Pharmacy, Shahid Beheshti University of Medical Sciences, Tehran, Iran; 3 School of Biotechnology, College of Science, University of Tehran, Tehran, Iran

**Keywords:** Formulation, Garlic, Ma’joun Soum, Organosulfur Compounds (OSCs), S-allyl Cysteine

## Abstract

**Background:**

Garlic (*Allium sativum*) is an important medicinal plant, and its therapeutic properties are primarily attributed to its organosulfur compounds (OSCs). However, the development of medicinal garlic-based formulations remains challenging because of the inherent instability of these bioactive compounds. Traditional garlic-based formulations may provide a suitable matrix for promoting the conversion of unstable sulfur compounds into more stable derivatives during aging.

**Objectives:**

This study investigated the time-dependent chemical transformations of major garlic-derived organosulfur compounds in Ma’joun Soum, a traditional Iranian garlic-based semisolid formulation prepared according to Qarabadin-e-Kabir. During a 30-day storage period, the effects of different aging processes on the concentrations of alliin, allicin, and S-allyl cysteine (SAC) were evaluated. A major strength of this study is the integration of a traditional pharmacopeial preparation with modern analytical characterization.

**Methods:**

Ma'joun Soum was prepared from fresh garlic, clarified cow butter (cow ghee), pasteurized milk, honey, and herbal ingredients according to the traditional pharmacopeial formulation. After preparation, samples were aged under three different conditions: room temperature (25°C), in a stability chamber (40°C), and under traditional underground aging conditions (33 - 36°C). The concentrations of alliin, allicin, and S-allyl cysteine (SAC) were quantified over time using high-performance liquid chromatography (HPLC), and sulfur-containing compounds were characterized using headspace gas chromatography-mass spectrometry (GC-MS).

**Results:**

Storage temperature and aging duration significantly influenced the chemical profile of Ma'joun Soum. Alliin exhibited a transient increase during the early stage of storage, followed by a gradual decline, whereas SAC progressively accumulated under all storage conditions. GC-MS analysis demonstrated a reduction in volatile sulfur-containing compounds during aging, while HPLC revealed compositional changes in selected water-soluble organosulfur compounds. These findings indicate that aging promotes complex chemical transformations within the formulation rather than simply preserving the original sulfur constituents.

**Conclusions:**

Short-term aging of Ma'joun Soum promotes the conversion of garlic-derived organosulfur compounds toward a composition enriched in stable, water-soluble sulfur metabolites, particularly SAC. Although the present study does not directly compare this formulation with conventional aged garlic extract (AGE), the findings suggest that controlled short-term aging may be a promising strategy for accelerating sulfur compound transformation in traditional garlic formulations.

## 1. Background

Garlic (*Allium sativum*), a well-known medicinal plant in the Amaryllidaceae family, is a bulbous flowering species widely used for culinary and therapeutic purposes. The medicinal value of garlic has been recognized for nearly 5,000 years, and it has played an important role in traditional Chinese medicine for at least 3,000 years (1). Numerous biological activities of garlic have been reported, including anticancer, antibacterial, antiviral, antidiabetic, antihypertensive, cardioprotective, hepatoprotective, lipid-lowering, and antioxidant effects. These pharmacological properties are primarily attributed to organosulfur compounds (OSCs) (2). Garlic also exhibits potent anti-inflammatory activity by reducing inflammatory biomarkers such as IL-6, CRP, and TNF-α. In addition, it enhances immune function by modulating cytokine production and stimulating immune cells, including natural killer (NK) cells (3). In athletes, garlic supplementation has demonstrated benefits in reducing exercise-induced oxidative stress and muscle damage, improving antioxidant capacity, and enhancing endurance performance (4).

Garlic-derived organosulfur compounds are generally categorized into oil- and water-soluble compounds (2). Alliin (S-allyl L-cysteine sulfoxide) is the predominant sulfur-containing compound in fresh garlic cloves. Upon crushing or cutting garlic, the enzyme alliinase is released and catalyzes the conversion of alliin to allicin (allyl 2-propenethiosulfinate). Allicin is responsible for the characteristic pungent odor of garlic and has limited water solubility. Although allicin is highly unstable, it readily decomposes into several oil-soluble sulfur compounds, mainly including dithiins, ajoene, allyl methyl trisulfide (AMTS), diallyl sulfide (DAS), diallyl disulfide (DADS), and diallyl trisulfide (DATS) (5, 6). In contrast, water-soluble organosulfur compounds, including S-allyl cysteine (SAC), S-allylmercaptocysteine (SAMC), and the metabolites allyl mercaptan (AM) and allyl methyl sulfide (AMS), are derived from γ-glutamyl-S-alk(en)yl-L-cysteines. These compounds have milder odors and less intense flavors than oil-soluble organosulfur compounds (7).

Consumption of fresh garlic may be associated with several adverse effects, including gastrointestinal disturbances, reduced erythrocyte counts, increased reticulocyte production, elevated bleeding risk, and gastric papilloma formation (8). Nevertheless, garlic-derived organosulfur compounds, including allicin, S-allyl cysteine (SAC), and S-allylmercaptocysteine (SAMC), are considered major contributors to the therapeutic properties of garlic, such as cardioprotective, anticancer, immunomodulatory, and antimicrobial activities (9). Despite their biological importance, many of these compounds are chemically unstable in fresh garlic. Therefore, different processing methods, particularly those involving controlled temperature and pharmaceutical formulations, may improve the chemical transformation of volatile organosulfur compounds. Aged garlic extract (AGE), typically produced through an aging process lasting up to 20 months, converts unstable compounds such as allicin into more stable and bioavailable antioxidants, including S-allyl cysteine and S-allylmercaptocysteine. This transformation enhances the antioxidant potential of AGE, enabling it to effectively scavenge reactive oxygen species (ROS), stimulate endogenous antioxidant enzymes, and protect against oxidative stress associated with aging.

Traditional multi-component herbal formulations, such as “Javarish” and “Ma’joun”, often incorporate aged garlic preparations to harness their therapeutic potential. These formulations have been investigated for their antioxidant, cardioprotective, immunomodulatory, and neuroprotective effects, supported by both experimental and clinical evidence. In traditional Persian medicine, Ma’joun refers to a compounded semisolid formulation prepared for various therapeutic purposes, including the elimination of bodily waste, stimulation of innate heat, strengthening of vital organs, and promotion of general well-being.

One of the most widely recognized garlic-based formulations in traditional medicine is “Ma’joun Soum”, which has also been described as an antidote for various types of poisoning. This formulation is referenced in historical texts under several names, including Dawai Nafia, Tirak al-Thum, and Ma’joun al-Nabi. Traditionally, Ma’joun Soum has been used for the management of conditions such as unilateral facial paralysis, tetany, conjunctival and corneal disorders, insect bites, seizures, muscle spasms, atherosclerosis, male sexual dysfunction, dizziness, and excessive phlegm accumulation. Traditional medical literature also emphasizes that the health-promoting effects of garlic depend on appropriate timing, individual temperament, and moderate consumption.

The preparation of Ma’joun Soum has been described in several traditional pharmaceutical texts, including Shafahi and Qarabadin Azam. In this study, Ma’joun Soum was prepared based on the Qarabadin-e-Kabir prescription and subsequently subjected to a defined aging period, traditionally referred to as the “perception time”, which is believed to promote the development of a secondary temperament and a more homogeneous formulation. It is hypothesized that this aging process facilitates changes in organosulfur compounds by converting allicin into more stable water-soluble organosulfur compounds, primarily SAC and SAMC, along with smaller quantities of oil-soluble allyl sulfides.

Similarly, aged garlic extract (AGE) is conventionally produced by aging garlic in a water and ethanol mixture at room temperature for more than 20 months, followed by filtration and concentration steps (10). In addition to organosulfur compounds, AGE contains several bioactive constituents, including phenolic compounds, flavonoids, pyruvate, and thiosulfate, which exhibit antioxidant, anti-inflammatory, and neuroprotective activities (11). Compared with oil-soluble OSCs, water-soluble OSCs demonstrate greater chemical stability and distinct pharmacokinetic characteristics in AGE (2). To shorten the aging duration while preserving product quality, several advanced processing strategies, such as controlled enzymatic treatment, high-temperature short-time aging, and modern stabilization techniques, may be employed. These approaches are intended to maintain the bioactive composition and antioxidant capacity of garlic preparations without requiring prolonged aging periods.

## 2. Objectives

The concentrations of alliin, allicin, and SAC were quantified using high-performance liquid chromatography (HPLC) during the time-course experiment, while changes in volatile organosulfur compounds were monitored to characterize chemical transformations occurring in garlic, the principal ingredient of Ma’joun Soum. Accordingly, the objective of this study was to investigate the effects of storage temperature and aging duration on the chemical transformation of major organosulfur compounds in Ma'joun Soum prepared according to Qarabadin-e-Kabir. Rather than demonstrating pharmaceutical stability or equivalence to conventional aged garlic extract, this study aimed to provide an analytical characterization of organosulfur compound transformations occurring during accelerated aging of a traditional garlic-based formulation.

## 3. Methods

In this study, Ma’joun Soum was prepared according to Qarabadin-e-Kabir, and the contents of organosulfur and terpenoid compounds were compared after 20 days of aging under three conditions: room temperature (25°C), stability chamber conditions (40°C), and traditional aging conditions (33 - 36°C).

### 3.1. Garlic Samples

Fresh garlic bulbs (*Allium sativum* L.) were collected from farms in Tuyserkan City, Hamedan Province, Iran, in June 2020. After harvest, healthy bulbs without visible mechanical damage or fungal infection were selected. A portion of the cloves was crushed and dried at room temperature for 1 week. Fresh crushed garlic was sampled after 24 h for subsequent analyses. After drying, the garlic was ground into powder. The remaining garlic samples were used to prepare Ma’joun Soum.

### 3.2. Ingredients of Ma’joun Soum

The formulation of Ma’joun Soum was selected from the Iranian traditional medicine textbook Qarabadin-e-Kabir under the monograph of Teriyaq al-Thum (Dawai Shafieh). The formulation included peeled garlic, clarified cow butter or cow ghee (containing > 99.5% milk fat, <0.5% moisture, a clear and golden-yellow semisolid fat at room temperature), pasteurized cow milk, honey, black cumin or black seeds (*Nigella sativa*), black pepper (*Piper nigrum*), and marjoram (*Origanum majorana*).

### 3.3. Preparation of Ma’joun Soum

Fresh garlic collected from Tuyserkan was peeled and crushed. The crushed garlic (420 g) was mixed with clarified cow butter (420 g; sourced from the Marivan region of Kurdistan) until the garlic surface was fully coated with oil. The mixture was heated at 70°C with continuous stirring for approximately 30 min, allowing gradual incorporation of the lipid phase and formation of a homogeneous mixture. The selected temperature and heating duration followed the traditional preparation procedure described in Qarabadin-e-Kabir and were intended to obtain a homogeneous semisolid formulation rather than to maximize enzymatic conversion of sulfur compounds. Because alliinase is known to undergo thermal inactivation at elevated temperatures, subsequent changes in organosulfur composition during storage were interpreted primarily as post-processing chemical transformations rather than continued enzymatic synthesis.

Next, pasteurized milk (Mahsham Co., 420 mL) was added, and heating at 70°C was continued for an additional 35 min until the aqueous phase was fully incorporated. According to the traditional recipe, honey was first heated at 60°C for 20 min until foaming occurred. Subsequently, 420 g of honey was blended with the other ingredients for 30 min at 60°C. After a uniform consistency was achieved, Ma’joun Soum was finalized by adding black seeds (32 g), black pepper (16 g), and marjoram (16 g). The entire preparation process was repeated three times for subsequent analyses.

### 3.4. Sampling of Ma’joun Soum

**Figure 1.**
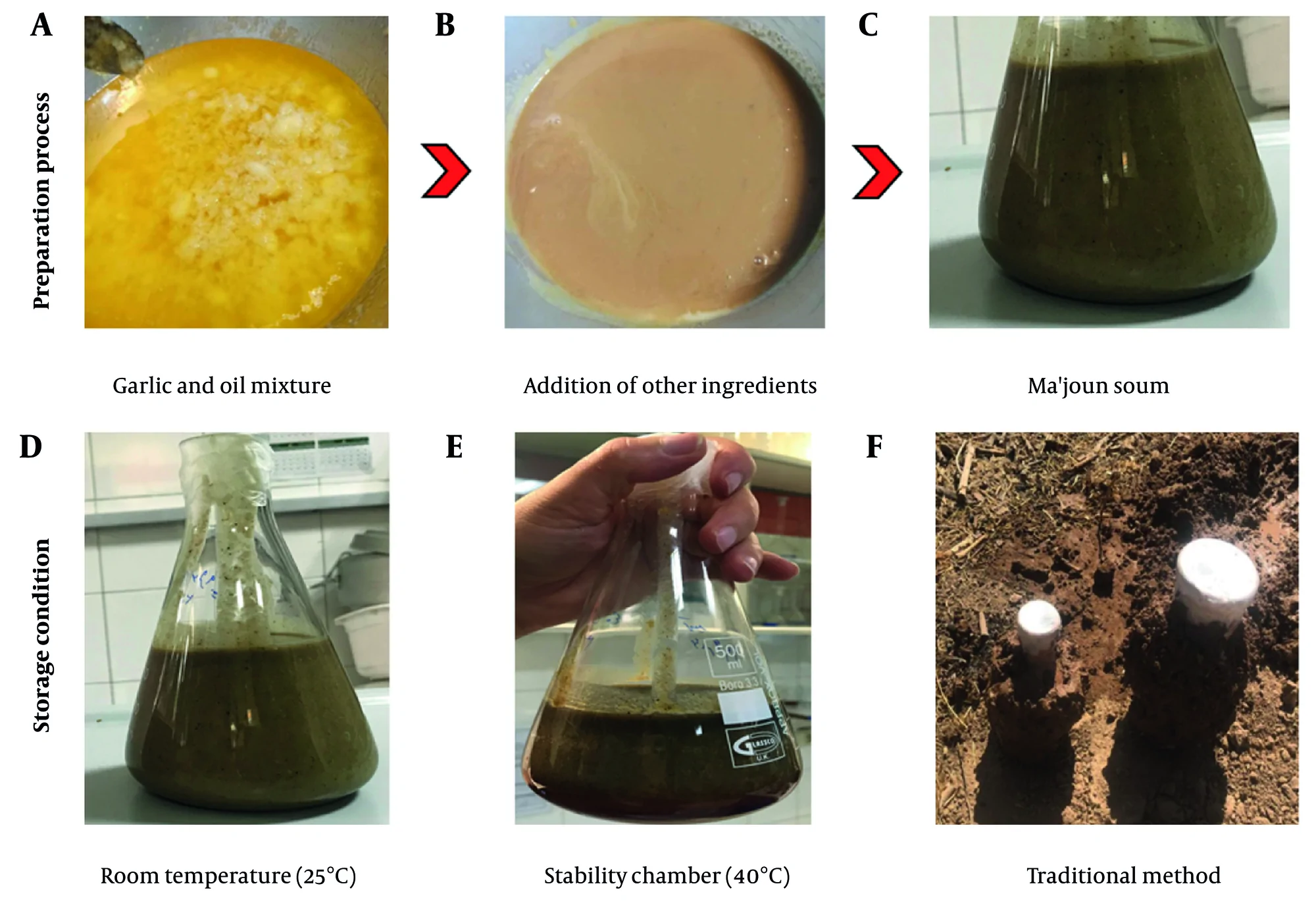
Preparation (A-C) and storage of Ma’joun Soum under (D) room temperature at 25°C, (E) stability testing chamber at 40 ± 2°C and 70% relative humidity, and (F) the traditional method.

After preparation of Ma’joun Soum (three batches; Figure 1A-C), samples from each batch were randomly divided into three groups and stored under different conditions: 1) The control group was stored at room temperature (25°C) (Figure 1D). 2) A second group was stored in a stability testing chamber at 40 ± 2°C with 70% relative humidity (Figure 1E). 3) For the traditional method, Ma’joun Soum was placed in a terracotta container that was greased with clarified cow butter and covered with cow dung. The container was buried in soil and maintained at 33 - 36°C (Figure 1F). Containers were buried at an approximate depth of 40 - 50 cm, where the soil temperature remained relatively constant throughout the experimental period. Temperature was monitored periodically using a calibrated digital thermometer inserted adjacent to the buried container. During the study, the soil temperature remained within 33 - 36°C, corresponding to the traditional maturation conditions described in Persian pharmacopeial literature. Sampling was performed in triplicate for each group and repeated from the same container during the time-course experiment at 0, 10, 20, and 30 days for metabolite analysis.

### 3.5. Profiling of Ma’joun Soum by GC-MS

Volatile compounds were analyzed using gas chromatography-mass spectrometry (GC-MS) coupled with a headspace sampler (Agilent Technologies 5977A-MSD, 7890B-GC, 7697A Headspace Sampler). Briefly, 300 mg of Ma’joun Soum was transferred into a 20 mL headspace vial for analysis. Under headspace sampling conditions, the oven, loop, and transfer line temperatures were set at 80°C, 90°C, and 100°C, respectively. Vial equilibration time, injection duration, and GC cycle time were 10 min, 1 min, and 60 min, respectively. Chromatographic separation was performed using an HP-5MS column (30 m × 0.25 mm ID × 0.25 µm film thickness).

For GC conditions, the oven temperature program was set from 60 to 210°C at a rate of 3°C/min, followed by an increase to 240°C at a rate of 20°C/min. The final temperature was held for 8.5 min. The total run time was 60 min. Mass spectrometric analysis was performed using electron ionization at 70 eV. The ion-source, interface line, and injector temperatures were maintained at 230°C, 280°C, and 280°C, respectively. The carrier gas was helium at a flow rate of 1 mL/min. The mass detector scanned a mass range of 50 - 480 m/z. Detected compounds were identified by comparing the obtained mass spectra with those in the NIST Mass Spectral Library database. Compounds with high similarity indices and match quality were tentatively identified. Retention times and relative peak area percentages were also recorded to characterize the phytochemical constituents.

### 3.6. Quantitative Determination of Allicin by HPLC

For sample preparation, 1.0 g of sample was mixed with 20.0 mL of water. The mixture was homogenized in an ultrasonic bath for 20 min and then kept at room temperature for 30 min. Samples were centrifuged at 3000 × g for 30 min, and 10.0 mL of the supernatant was diluted to 25 mL using solution A, consisting of methanol/water containing 1% formic acid (60/40, v/v). The mixture was shaken and centrifuged again at 3000 × g for 5 min. Next, 0.5 mL of the internal standard solution was transferred into a volumetric flask and diluted to 10.0 mL with solution A. The final solution was filtered through a 0.2 μm syringe filter before injection. The internal standard solution was prepared by dissolving 20.0 mg of butylparahydroxybenzoate in 100.0 mL of methanol and water (1:1, v/v).

**Figure 2.**
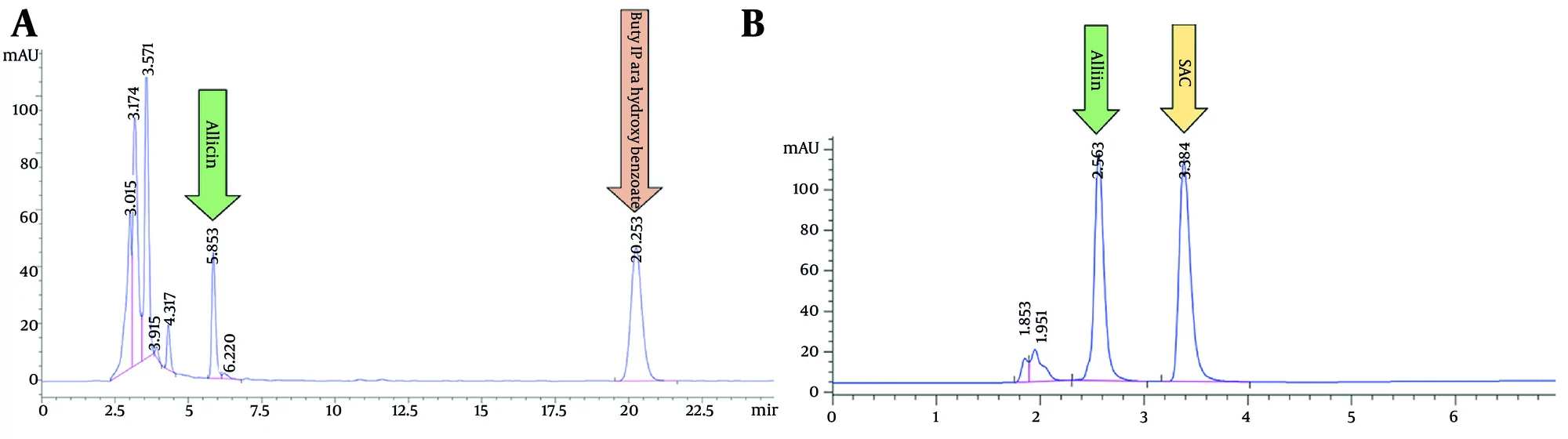
High-performance liquid chromatography (HPLC) chromatogram of (A) allicin and butylparahydroxybenzoate as standards in garlic and (B) calibration curves of alliin and S-allyl cysteine (SAC) as standards.

Allicin quantification was performed using an Agilent HPLC system equipped with a photodiode array detector (scan range: 190 - 400 nm). HPLC conditions were as follows: Agilent Zorbax HPLC C18 column (5 μm, 4.6 × 250 mm), column temperature of 25°C; flow rate of 0.8 mL/min; and mobile phase consisting of methanol and water containing 0.1% formic acid (60:40, v/v). The injection volume was 20 μL, and the run time was 25 min (Figure 2A). Quantitative analysis was performed at a detection wavelength of 254 nm. The percentage of allicin was calculated using the following equation (12):

$$\mathrm{Allicin}\left(\%\right)=\frac{S1\times M2\times 22.75}{S2\times M1}$$

Where:

S1 = peak area corresponding to allicin in the chromatogram of the test solution;

S2 = peak area corresponding to butyl parahydroxybenzoate in the chromatogram of the test solution;

M1 = mass of the analyzed sample (g);

M2 = mass of butyl parahydroxybenzoate in 100.0 mL of the internal standard solution (g).

Chromatographic specificity was demonstrated by complete baseline separation of allicin (retention time 5.85 min) from the internal standard (butyl parahydroxybenzoate, retention time 20.25 min) and other endogenous matrix components. No interfering peaks were observed at the retention time of allicin in blank matrix samples, confirming adequate specificity for quantitative analysis.

### 3.7. Quantitative Determination of Alliin and S-Allyl Cysteine by HPLC

For extraction, 1 g of sample was mixed with 2.5 mL of solution A, containing 50 μL HCl in 50 mL methanol. The mixture was vortexed thoroughly for 5 min. Subsequently, 4 mL of solution A was added, and the mixture was vortexed again for 5 min. The solution was filtered using Whatman filter paper, and an additional 3 mL of solution A was used to wash the filter paper. The filtrate volume was adjusted to 10 mL with solution A. After final filtration, the sample was injected into the HPLC system.

For quantitative analysis, L-(+)-alliin and S-allyl-L-cysteine (SAC) were used as external standards to generate calibration curves. Detection was performed at 210 nm. The mobile phase consisted of methanol/water (30/70, v/v) containing sodium dodecyl sulfate (SDS) (0.05% w/v), at a flow rate of 1.0 mL/min. Separation was performed using an Agilent Zorbax C18 column (5 μm, 4.6 × 250 mm) maintained at 25°C (Figure 2B). The calibration curves for SAC and alliin were established using standard concentrations ranging from 1 - 10 ppm and 0.5 - 1000 ppm, respectively (Figure 2B). A strong linear relationship between concentration and HPLC peak area was observed, with regression coefficients (R^2^) of 0.9901 for SAC and 0.9795 for alliin. The limits of detection (LOD) and quantification (LOQ) were determined according to International Council for Harmonisation (ICH) guidelines based on the standard deviation of the response and the slope of the calibration curves. The calculated LOD and LOQ values for SAC were 1.34 ppm and 4.07 ppm, respectively, whereas the corresponding values for alliin were 80.5 ppm and 244.1 ppm, respectively, indicating acceptable analytical sensitivity for quantification of both compounds.

### 3.8. Statistical Analyses

All data were analyzed by one-way ANOVA followed by Tukey's multiple comparison test using GraphPad Prism software version 8 (**** means P < 0.0001, *** means P < 0.001, ** means P < 0.01, * means P < 0.05). All experiments were performed in three independent replicates (three biological/formulation batches) per time point, and the results were expressed as means ± standard errors.

## 4. Results

### 4.1. Changes in Organosulfur Compounds in Fresh and Dried Garlic

**Figure 3.**
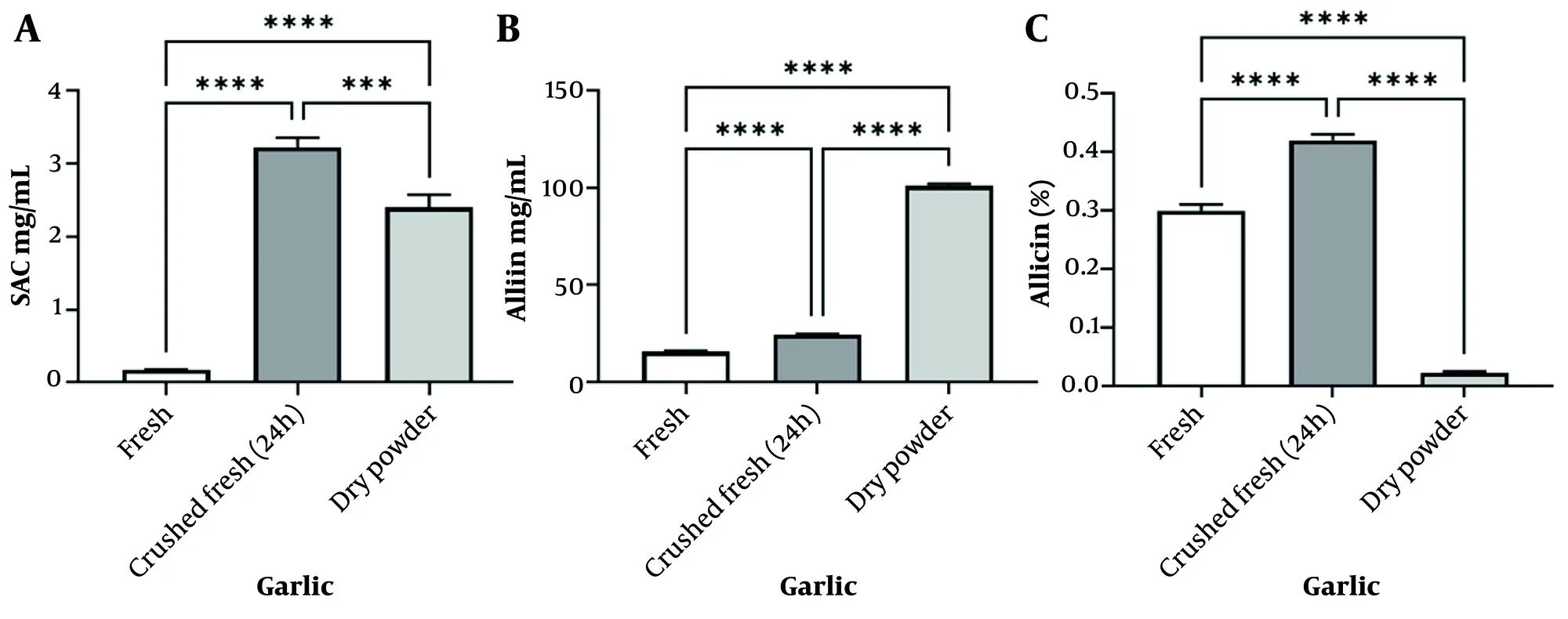
Changes in (A) SAC, (B) alliin, and (C) allicin content in fresh garlic, crushed fresh garlic, and dried garlic powder. Data represent mean values and standard errors from three independent experimental series (three biological/formulation batches) per time point; error bars represent standard error. *** and **** indicate statistically significant differences at P < 0.001 and P < 0.0001, respectively, by one-way ANOVA with Tukey multiple-comparison test. SAC: S-allyl cysteine.

To evaluate changes in organosulfur compounds in fresh garlic and garlic powder, we crushed fresh garlic, kept it at room temperature for 24 h, and compared it with fresh garlic at 0 h as the control. Three major organosulfur metabolites involved in garlic sulfur metabolism, including SAC, alliin, and allicin, were quantified using HPLC. The measured concentrations of all three compounds increased significantly in crushed fresh garlic after 24 h compared with the control samples (Figure 3A-C). As shown in Figure 3A, SAC content in fresh garlic was approximately 1.7 µg/g, whereas crushing and incubation for 24 h significantly increased the SAC concentration to nearly 32 µg/g. In dried garlic powder, SAC content slightly decreased compared with crushed fresh garlic but remained significantly elevated at approximately 24 µg/g relative to fresh garlic. Similarly, alliin content in fresh garlic was 0.15 mg/g, whereas crushing and incubation for 24 h resulted in a substantial increase to 0.24 mg/g, representing approximately a 1.55-fold elevation compared with fresh garlic (Figure 3B). In dried garlic powder, alliin reached 1.01 mg/g, corresponding to an approximately 6.3-fold increase relative to fresh garlic and a 4.1-fold increase compared with crushed fresh garlic (Figure 3B). The measured (recoverable) allicin content varied substantially across processing conditions (Figure 3C). Fresh garlic exhibited a mean value of 0.30%, which increased markedly following crushing and 24 h incubation (0.42%), indicating enhanced formation under tissue disruption conditions. In contrast, drying and powder processing resulted in a dramatic reduction in allicin content (0.02%), approaching the basal levels observed in fresh garlic. These results demonstrate that mechanical disruption promotes allicin formation, whereas dehydration processing markedly diminishes its content (Figure 3C).

### 4.2. Profiling of Volatile Compounds in Ma’joun Soum

**Figure 4.**
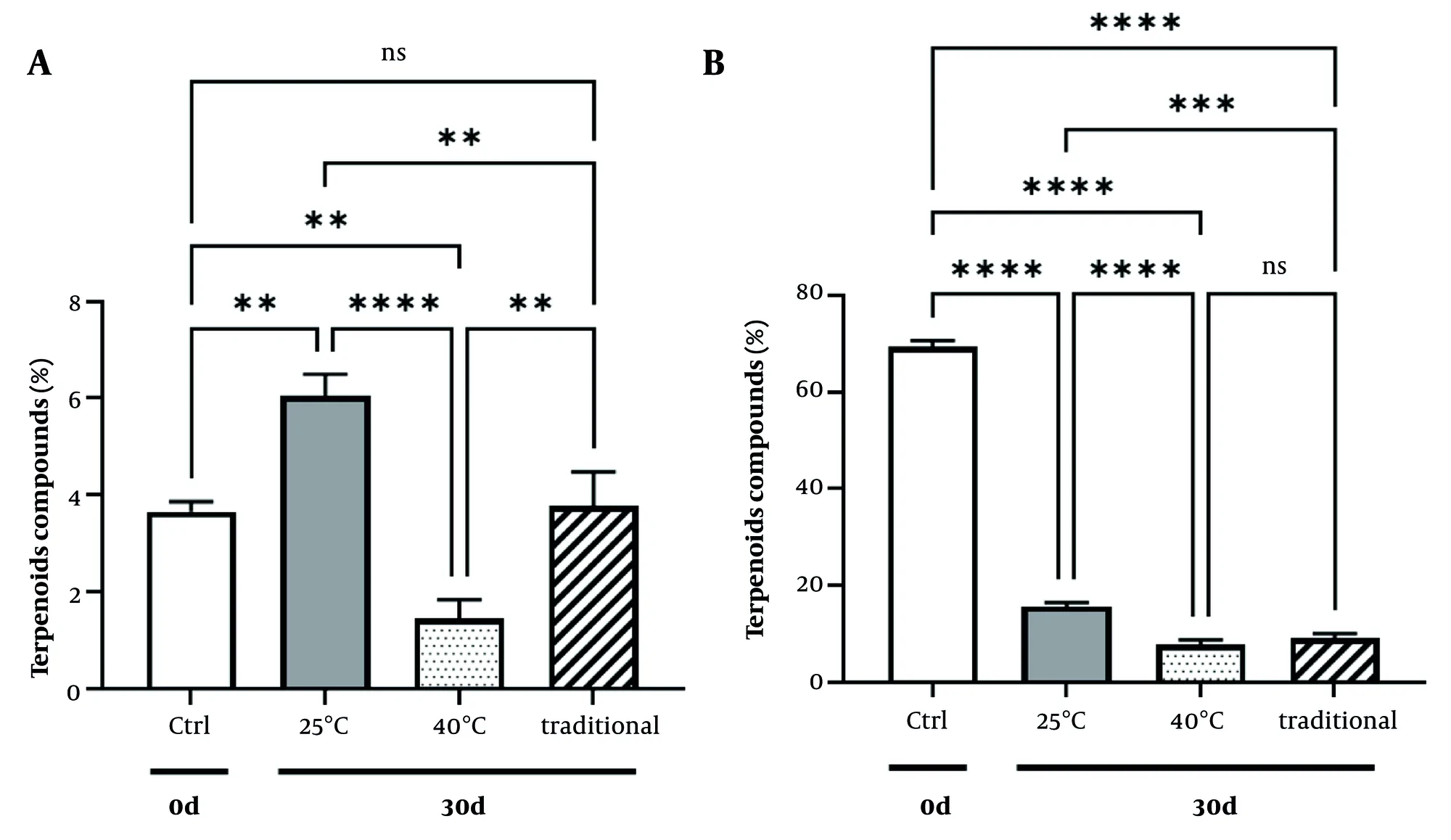
Content of (A) terpenoids and (B) organosulfur compounds in Ma’joun Soum. Samples were stored under different conditions, including room temperature (25°C), a stability testing chamber (40°C), and the traditional method, for 20 d and compared with the control at 0 d. Data represent mean values and standard errors from three independent experimental series (three biological/formulation batches) per time point; error bars represent standard error. **, ***, and **** indicate statistically significant differences at P < 0.01, P < 0.001, and P < 0.0001, respectively, by one-way ANOVA with Tukey multiple-comparison test. ns, nonsignificant.

The volatile composition of Ma’joun Soum was investigated under different storage and processing conditions, including room temperature, stability chamber conditions, and the traditional preparation method. Volatile metabolites were analyzed after 20 d of storage using GC-MS. Based on their chemical characteristics, the identified compounds were categorized into two groups, including terpenoids and sulfur-containing compounds (Figure 4A, B). The results indicated that terpenoid compounds increased in samples stored at room temperature, whereas their levels decreased under stability chamber conditions after 20 d. In contrast, no substantial changes were observed in samples prepared using the traditional method compared with the control samples at 0 d (Figure 4A). Compared with the control, sulfur-containing compounds significantly decreased under all evaluated conditions after 20 d of storage (Figure 4B). As summarized in Table 1, GC–MS analysis revealed the presence of several phytochemical constituents, predominantly organic sulfur derivatives and terpenoid compounds. Samples processed by the traditional method or maintained in the stability chamber exhibited a more pronounced reduction in sulfur-containing compounds than samples stored at room temperature after 20 d (Figure 4B). Metabolite identification was performed by comparing mass fragmentation patterns with spectra available in the NIST Mass Spectral Library database. Overall, the results indicated that organic sulfur-containing derivatives represented a major fraction of the detected metabolites, alongside various terpenoid compounds (Table 1).

**Table 1.** GC-MS-Based Identification of Organic Sulfur Derivatives and Terpenoids in Ma’joun Soum Using the NIST Mass Spectral Library

Ma’joun Soum/Control and Compound Names	Probability (%)	Percentage (%)	Ma’joun Soum/40°C and Compound Name	Probability (%)	Percentage (%)
**Organic sulfur derivatives ^a^**			Organic sulfur derivatives ^b^		
Disulfide, dimethyl	94	3.850	Diallyl sulfide	95	0.227
Disulfide, methyl 2-propenyl	95	5.869	Disulfide, methyl 2-propenyl	96	0.823
(Z)-1-Methyl-2-(prop-1-en-1-yl)disulfane	95	1.159	Dimethyl trisulfide	93	0.614
(E)-1-Methyl-2-(prop-1-en-1-yl)disulfane	97	12.896	Trisulfide, di-2-propenyl	64	0.344
Dimethyl trisulfide	96	4.194	Diallyl disulfide	87	1.876
Diallyl disulfide	90	2.454	(E)-1-Allyl-2-(prop-1-en-1-yl)disulfane	95	0.465
(Z)-1-Allyl-2-(prop-1-en-1-yl)disulfane	96	5.435	Trisulfide, methyl 2-propenyl	96	1.175
Di-tert-butyl disulfide	87	4.174	3-Vinyl-1,2-dithiacyclohex-4-ene	97	1.459
Trisulfide, methyl 2-propenyl	91	4.804	2-Vinyl-4H-1,3-dithiine	98	0.815
3-Vinyl-1,2-dithiacyclohex-4-ene	97	12.935	Monoterpene ^c^		
2-Vinyl-4H-1,3-dithiine	98	10.763	Thymoquinone	27	0.034
Trisulfide, di-2-propenyl	59	0.798	3-Carene	89	0.158
**Monoterpene ^c^**			Cyclohexene, 1-methyl-4-(1-methylethylidene)-	90	0.102
o-Cymene	87	0.782	p-Cymene	95	0.510
γ-Terpinene	60	0.410	D-Limonene	93	0.136
Thymoquinone	93	0.442	γ-Terpinene	93	0.233
1,4-Cyclohexadiene, 1-methyl-4-(1-methylethyl)-	90	2.033	Cyclohexane, 1-methylene-4-(1-methylethenyl)-	96	0.128
-	-	-	Bicyclo[7.2.0]undec-4-ene, 4,11,11-trimethyl-8-methylene-, (E)-(1R,9S)-(-)-	99	0.138

^a^ Sum of organic sulfur derivatives is 69.331%.

^b^ Sum of organic sulfur derivatives is 7.798%.

^c^ Sum of terpenoids is 3.667%.

### 4.3. Allicin Increased in Ma’joun Soum in a Time-Dependent Manner

**Figure 5.**
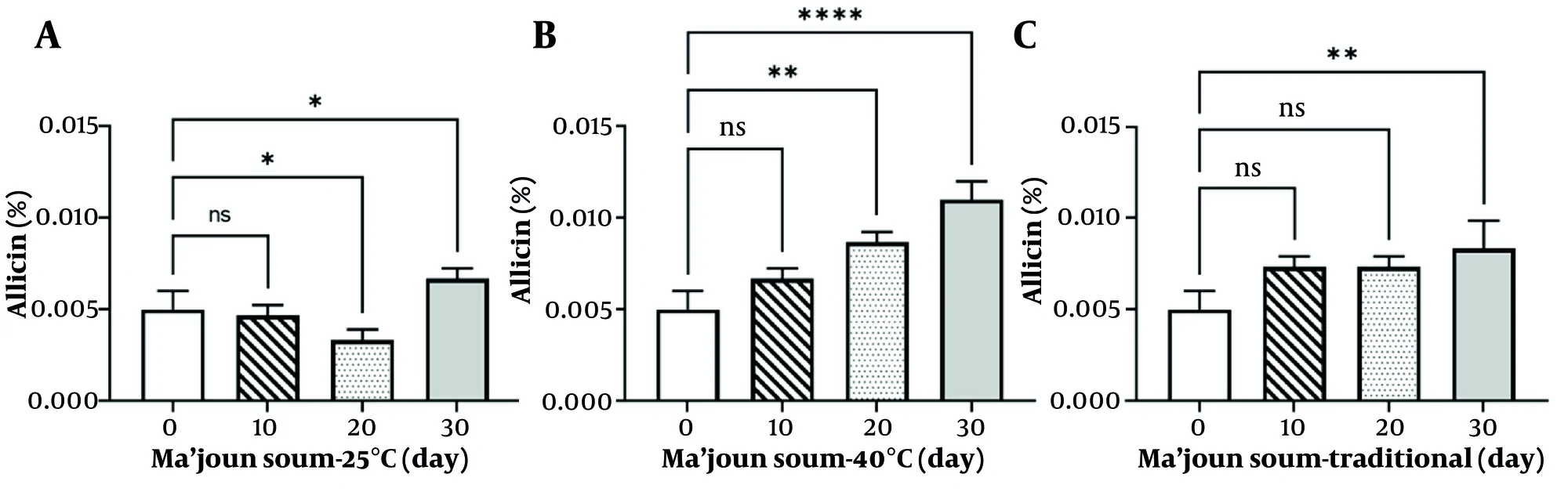
Analysis of allicin content in Ma’joun Soum over time. Samples were stored under (A) room temperature (25°C), (B) stability testing chamber conditions (40°C), and (C) the traditional method at 0, 10, 20, and 30 d. Data represent mean values and standard errors from three independent experimental series (three biological/formulation batches) per time point; error bars represent standard error. *, **, and **** indicate statistically significant differences at P < 0.05, P < 0.01, and P < 0.0001, respectively, by one-way ANOVA with Tukey multiple-comparison test. ns, nonsignificant.

To determine the allicin content during storage of Ma’joun Soum, samples were collected over a defined time course and analyzed using HPLC. The results indicated that the measured (recoverable) allicin content increased significantly after 30 d of storage under all tested conditions, including room temperature (0.006%; 1.3-fold), stability testing chamber conditions (0.01%; 2.2-fold), and the traditional preparation method (0.008%; 1.7-fold), compared with the initial control samples at 0 d (Figure 5A-C).

Under room temperature conditions, the allicin content decreased until day 20 and subsequently increased at day 30 (Figure 5A). In samples stored in the stability testing chamber, allicin retention/recovery exhibited a continuous upward trend throughout the storage period, with a significant increase observed between days 20 and 30 relative to 0 d (Figure 5B). Similarly, the pattern of allicin retention/recovery in samples prepared using the traditional method showed an increasing trend during the storage period (Figure 5C).

### 4.4. Alliin Exhibited Transient Accumulation Followed by Gradual Decline During Aging

**Figure 6.**
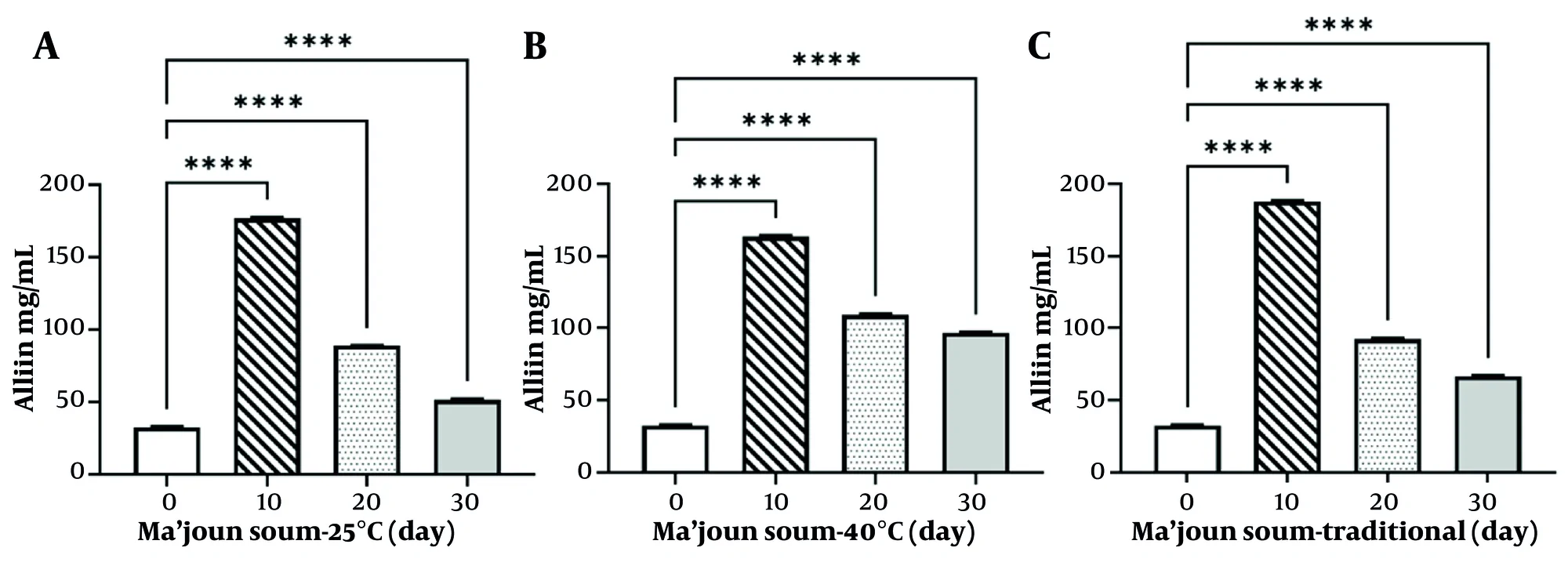
Analysis of alliin content in Ma’joun Soum over time. Samples were stored under (A) room temperature (25°C), (B) stability testing chamber conditions (40°C), and (C) the traditional method at 0, 10, 20, and 30 d. Data represent mean values and standard errors from three independent experimental series (three biological/formulation batches) per time point; error bars represent standard error. *, **, and **** indicate statistically significant differences at P < 0.05, P < 0.01, and P < 0.0001, respectively, by one-way ANOVA with Tukey multiple-comparison test.

Alliin, a major natural sulfur-containing constituent of garlic and the precursor of allicin through alliinase activity, was also evaluated during storage. The results indicated that alliin content first increased and then decreased over the storage period under all experimental conditions (Table 2). Overall, compared with the initial level at 0 d, alliin concentration increased more than 5-fold after 10 d of storage in samples maintained at room temperature (5.4-fold), in the stability chamber (5-fold), and under the traditional preparation method (5.7-fold) (Figure 6A-C). Following day 10, alliin levels gradually declined until day 30; however, the concentrations remained higher than the baseline values measured at 0 d. These findings indicate that alliin exhibited a transient accumulation pattern followed by a gradual reduction during prolonged storage.

**Table 2.** Concentrations of Alliin and SAC in Ma’joun Soum at Different Time Points (0, 10, 20, and 30 D) Under Room Temperature (25°C), Stability Chamber (40°C), and Traditional Aging Conditions ^a^

Compounds and Conditions	0 (d)	10 (d)	20 (d)	30 (d)
**Alliin (mg/g Ma’joun Soum)**	0.32 ± 0.005			
25°C		1.79 ± 0.009	0.89 ± 0.002	0.51 ± 0.007
40°C		1.63 ± 0.009	1.091 ± 0.009	0.97 ± 0.009
Traditional		1.87 ± 0.009	0.92 ± 0.008	0.66 ± 0.007
**SAC (μg/g Ma’joun Soum)**	19 ± 1.00			
25°C		74 ± 2.00	95.93 ± 1.95	159.5 ± 2.50
40°C		19.4 ± 1.00	42.63 ± 0.90	173.7 ± 1.57
Traditional		66 ± 4.63	82.367 ± 2.50	137.5 ± 2.29

^a^ Values are expressed as mean ± SD. n = 3.

### 4.5. S-Allyl Cysteine Increased With Longer Storage Duration

**Figure 7.**
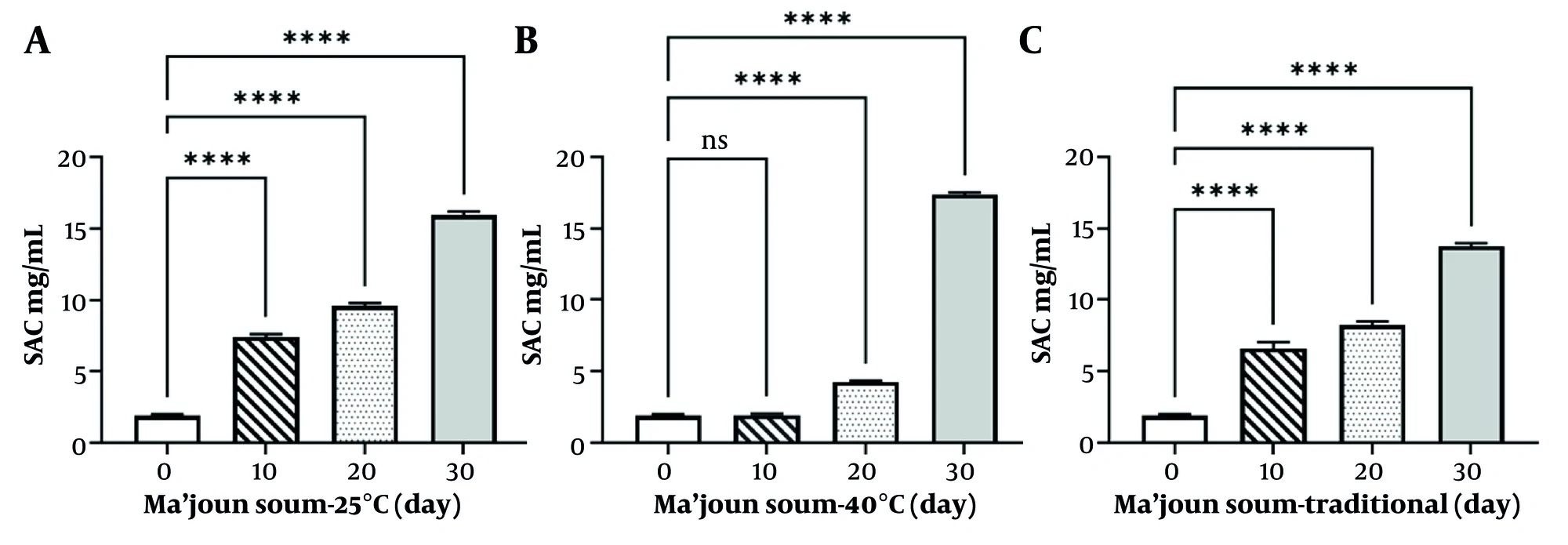
Analysis of SAC content in Ma’joun Soum over time. Samples were stored under (A) room temperature (25°C), (B) stability testing chamber conditions (40°C), and (C) the traditional method at 0, 10, 20, and 30 d. Data represent mean values and standard errors from three independent experimental series per time point; error bars represent standard error. **** indicates statistically significant differences at P < 0.0001 by one-way ANOVA with Tukey multiple-comparison test. ns, nonsignificant. SAC: S-allyl cysteine.

In parallel with allicin retention/recovery, we investigated the measured content of SAC, a water-soluble constituent in the organosulfur metabolic pathway, during storage of Ma’joun Soum. The results revealed a progressive increase in SAC accumulation over time under all evaluated storage conditions (Table 2). Relative to the control samples at 0 d (19 µg/g), SAC levels increased by 8.3-fold (159.50 µg/g), 9.1-fold (173.7 µg/g), and 7.2-fold (137.5 µg/g) after 30 d of storage under room temperature, stability chamber conditions, and the traditional preparation method, respectively (Figure 7A-C). These findings suggest that prolonged storage promotes the accumulation of SAC in Ma’joun Soum irrespective of the storage condition used.

## 5. Discussion

In the present study, it was hypothesized that the traditional semisolid formulation (Ma’joun Soum) undergoes chemical transformations during the “perception time” aging process, leading to compositional changes in organosulfur compounds and enhancement of its therapeutic potential. A similar phenomenon has been reported for aged garlic extract (AGE), in which specific processing conditions promote the chemical transformation of organosulfur compounds and improve the biological activity of the final product (13, 7). Conventionally, AGE is prepared by aging sliced garlic cloves in a water-ethanol solution at room temperature for more than 20 months (13). Following filtration and concentration, the final extract contains higher levels of stable and less odorous sulfur compounds due to the gradual conversion of allicin into more stable derivatives. Allicin, which is produced through alliinase-mediated conversion of alliin after garlic tissue disruption, is primarily responsible for the characteristic pungent odor and flavor of fresh garlic and serves as a precursor for several biologically active sulfur-containing compounds (9). Our proposed process may offer a shorter approach for producing a garlic-based formulation enriched in selected organosulfur compounds. However, direct comparison with conventional aged garlic extract is needed to establish equivalence.

Traditional Persian medicine texts, including Makhzan al-Adwieh, also emphasize that the therapeutic effects of garlic depend on appropriate timing, individual temperament, and moderate consumption, which are believed to preserve health and reduce harmful effects. These traditional concepts may indirectly reflect the importance of preparation methods and aging conditions in modulating the chemical composition and medicinal properties of garlic-based formulations. Furthermore, variations in garlic cultivar, geographical origin, and environmental growing conditions can substantially influence the metabolite composition and organosulfur profile of garlic, thereby affecting the chemical characteristics and biological activity of aged garlic products (14). Previous studies have demonstrated that different *Allium* species exhibit distinct pharmacological activities associated with their organosulfur constituents. For example, *Allium jesdianum* has shown significant anticancer activity against melanoma cells by inducing apoptosis and oxidative stress pathways, while exhibiting low toxicity toward normal cells (15). These findings further support the pharmacological potential of organosulfur-rich *Allium* species and their bioactive compounds.

**Figure 8.**
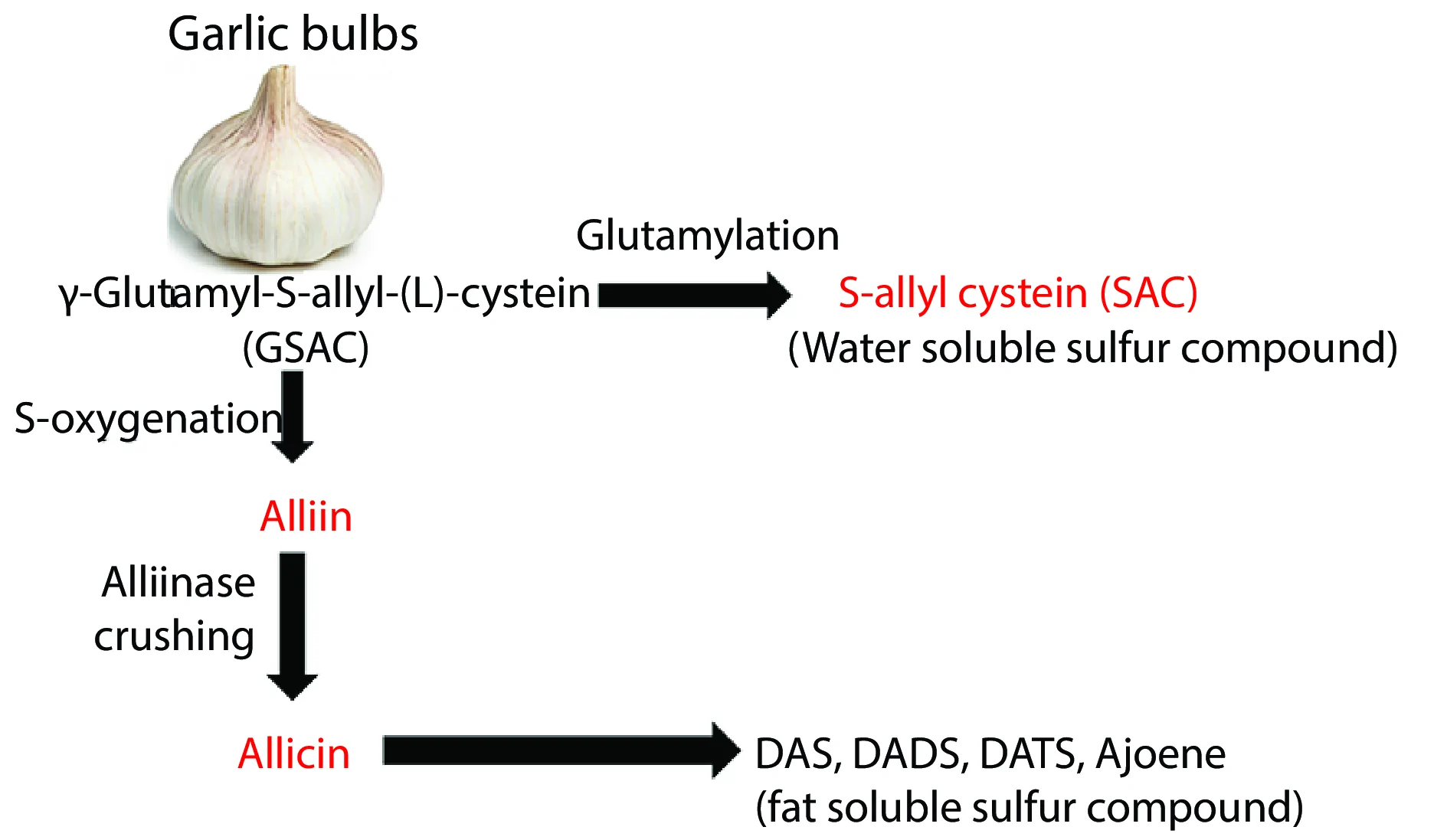
Organosulfur compounds in garlic. The primary sulfur compounds are γ-glutamyl-S-alk(en)yl-L-cysteines, which serve as substrates in two pathways: 1) production of water-soluble compounds, mainly SAC and S-allylmercaptocysteine (SAMC), and 2) production of alk(en)yl-L-cysteine sulfoxide (alliin) through oxidation. Allicin is produced from alliin by the vacuolar enzyme alliinase when garlic is crushed. Allicin rapidly decomposes to diallyl disulfide (DADS), diallyl sulfide (DAS), diallyl trisulfide (DATS), vinyl dithiin, and ajoene, which are oil-soluble compounds. SAC: S-allyl cysteine.

As shown in Figure 8, γ-glutamyl cysteine serves as the primary precursor for the formation of sulfur-containing amino acid derivatives in fresh garlic cloves, predominantly alliin (S-allyl L-cysteine sulfoxide), followed by methiin (S-methylcysteine sulfoxide) and isoalliin. Upon crushing or cutting garlic tissue, the enzyme alliinase catalyzes the conversion of alliin into allicin (6). Because alliinase is sensitive to acidic conditions, the alliin-alliinase system exhibits optimal activity at approximately 33°C and pH 6.5 in the presence of water (16). The results of the present study demonstrated that prolonged storage reduced alliin accumulation in a time-dependent manner; however, the rate of decline was strongly influenced by temperature. Stability chambers were used to evaluate the time-dependent stability of the formulation under different storage conditions. Under the traditional aging condition (33 - 36°C), the reduction in alliin content was more pronounced than that observed in the 40°C stability chamber, particularly between days 10 and 30. The transient increase in measured alliin during the initial storage period is likely attributable to enhanced extraction and progressive release of residual intracellular alliin from partially disrupted garlic tissues rather than de novo biosynthesis. In the non-living Ma'joun Soum formulation, no metabolic pathway exists to regenerate alliin after processing. The subsequent decrease or stabilization of alliin during extended storage is consistent with the gradual chemical transformation and degradation of garlic organosulfur compounds. Previous studies have also shown that temperature and processing duration critically affect the composition of garlic-derived bioactive compounds. For example, the optimal extraction of total polyphenols, flavonoids, thiosulfinates, and anthocyanins from dried garlic husk has been reported at temperatures between 60 and 70°C with extraction times ranging from 60 to 90 min (17). However, certain sulfur-containing compounds are generated only under specific heating conditions and subsequently degrade upon prolonged exposure. For example, 3-(allyltrisulfanyl)-2-aminopropanoic acid, a slightly water-soluble antimicrobial sulfur compound, reached its maximum concentration after heating at 120°C for 30 min and disappeared completely after 90 min of heating (18). Such transformations may potentially be optimized through advanced processing approaches, including high-temperature short-time (HTST) treatment, enzymatic processing, and modern extraction techniques such as microwave-assisted or ultrasound-assisted extraction.

Allicin is abundantly produced through alliinase-mediated reactions and accounts for approximately 70% of the initial thiosulfinate content in crushed garlic (7). Due to its low water solubility and high chemical instability, allicin is rapidly converted into oil-soluble sulfur compounds, including dithiins, diallylsulfide (DAS), diallyl disulfide (DADS), diallyl trisulfide (DATS), and ajoene. These degradation processes may occur within hours at room temperature and within minutes during thermal cooking. *Allium* species constitute a rich source of natural antifungal agents, with allicin, ajoene, and steroidal saponins being the most promising compounds. Their multitarget mechanisms, broad-spectrum antifungal activity, and potential synergy with conventional antifungal drugs make them attractive candidates for future pharmaceutical, agricultural, and food-industry applications (19). In another study, garlic-derived compounds were proposed to modulate host immune responses through regulation of inflammatory signaling pathways and activation of antiviral defense mechanisms (20). The antioxidant capacity of AGE is largely attributed to the high bioavailability of water-soluble organosulfur compounds, particularly SAC and SAMC, which are rapidly absorbed through the intestinal tract (21).

In the present study, SAC accumulation occurred more rapidly during the first 10 days under both the traditional aging condition and room temperature than under the stability chamber condition. Moreover, SAC concentrations increased progressively over the 30-day storage period under all experimental conditions. The observed increase in the measured SAC concentration during storage is therefore interpreted as an increase in its recoverable amount rather than new biosynthesis. SAC is generally considered to originate from the gradual conversion of naturally occurring γ-glutamyl-S-allyl-L-cysteine and related γ-glutamyl peptides present in garlic. In addition, the lipid-rich ghee matrix and storage conditions may facilitate the gradual release of SAC and its precursors from the garlic tissue, thereby improving extraction efficiency and analytical recovery. SAC, a major bioactive constituent of aged garlic extract (AGE), is a stable, water-soluble, and highly bioavailable organosulfur compound with well-established antioxidant and cytoprotective properties. Unlike oil-soluble organosulfur compounds, which are poorly detectable after garlic consumption, SAC is readily absorbed and detected in plasma, making it one of the principal contributors to the biological effects of AGE.

Currently, commercially available AGE-based pharmaceutical products, such as Kyolic products, are standardized according to SAC content. Clinical studies have shown that these products can improve endothelial function and reduce inflammatory markers in patients with metabolic syndrome, thereby slowing the progression of endothelial dysfunction (10). Furthermore, supplementation with a Kyolic capsule containing SAC has been associated with significant reductions in blood pressure compared with placebo treatment (22). Because the stability of water-soluble OSCs such as SAC and SAMC is influenced by factors including light exposure, oxygen availability, temperature, and processing conditions (2), semisolid formulations such as electuaries may represent suitable delivery systems for enhancing SAC accumulation during storage. In particular, the traditional preparation methods of Ma’joun Soum may provide favorable conditions for the compositional transformation of water-soluble organosulfur compounds. In future studies, encapsulation technologies and other advanced preservation approaches could further improve the bioavailability of garlic-derived OSCs for nutritional and therapeutic applications.

This study provides a foundation for future investigations of the biological and therapeutic potential of the accelerated formulation enriched in S-allyl cysteine (SAC). Future studies can further strengthen these findings by evaluating the biological activity, pharmacological properties, and clinical efficacy of the formulation. In addition, assessment of SAC bioavailability, metabolism, and systemic exposure will help clarify its physiological relevance. Direct measurement of antioxidant activity using standard assays could also provide valuable functional insight. Finally, comprehensive characterization of other garlic-derived organosulfur compounds may contribute to a better understanding of the overall bioactive profile of the accelerated aged garlic extract.

### 5.1. Conclusions

Ma’joun Soum was formulated according to the Qarabadin-e-Kabir pharmacopeial text using garlic extract as the principal component and was prepared within a substantially shorter timeframe. In the present study, the effects of storage duration on Ma’joun Soum were evaluated by monitoring changes in organosulfur compounds over 30 days under three different conditions: room temperature, stability chamber conditions, and traditional aging conditions. Because allicin is responsible for the pungent and unpleasant flavor of fresh garlic, reducing its concentration while increasing chemical transformation may improve consumer acceptability. The results demonstrated that aging of garlic-containing mixtures promoted the accumulation of SAC, a water-soluble bioactive OSC with a milder flavor profile than oil-soluble OSCs. In addition, the observed decline in alliin during storage is consistent with post-processing chemical transformation and degradation of garlic organosulfur compounds after thermal preparation. These findings suggest that accelerated aging approaches may facilitate the conversion of unstable sulfur compounds into more stable and bioavailable derivatives within a shorter processing period. Future studies should evaluate the bioavailability, absorption, metabolism, and systemic exposure of SAC, as a higher SAC concentration may not necessarily correspond to greater physiological efficacy in vivo.

## Data Availability

The data presented in this study are uploaded during submission and are openly available for readers upon request.
